# How Plants Tolerate Salt Stress

**DOI:** 10.3390/cimb45070374

**Published:** 2023-07-15

**Authors:** Haiqi Fu, Yongqing Yang

**Affiliations:** 1 State Key Laboratory of Plant Physiology and Biochemistry, College of Biological Sciences, China Agricultural University, Beijing 100193, China; fuhaiqi00000@126.com; 2 Tianjin Key Laboratory of Crop Genetics and Breeding, Institute of Crop Sciences, Tianjin Academy of Agricultural Sciences, Tianjin 300380, China

**Keywords:** salt stress, osmotic stress, oxidative stress, ionic stress, SOS pathway, phytohormone

## Abstract

Soil salinization inhibits plant growth and seriously restricts food security and agricultural development. Excessive salt can cause ionic stress, osmotic stress, and ultimately oxidative stress in plants. Plants exclude excess salt from their cells to help maintain ionic homeostasis and stimulate phytohormone signaling pathways, thereby balancing growth and stress tolerance to enhance their survival. Continuous innovations in scientific research techniques have allowed great strides in understanding how plants actively resist salt stress. Here, we briefly summarize recent achievements in elucidating ionic homeostasis, osmotic stress regulation, oxidative stress regulation, and plant hormonal responses under salt stress. Such achievements lay the foundation for a comprehensive understanding of plant salt-tolerance mechanisms.

## 1. Introduction

Soil salinization is a major adverse environmental stress that seriously restricts plant growth and development and affects crop yields [1]. Globally, one-third of agricultural land (~950 million hectares worldwide, including 250 million hectares of irrigated land) is affected by salt stress [2]. Plants are classified as halophytic or glycophytic according to their salt tolerance. Most crops are glycophytic and sensitive to high salt concentrations in the soil. Improper use of fertilizers, excess irrigation, and industrial pollution are the primary causes of widespread soil salinity [3,4], which poses a serious threat to agricultural productivity and food security for both humans and livestock. Developing crops that can grow normally in salinized soils is fundamental to solving this problem.

The basic mechanisms of plant salt tolerance have been well studied through the continuous development of research methods and technological innovations and the unremitting efforts of researchers. Many resulting findings elucidating how plants resist and adapt to salt stress at different levels have been applied to agricultural production. High concentrations of Na^+^ and Cl^−^ in the soil result in both osmotic and ionic stress, which decrease plants’ capacity to take up water and nutrients [5,6,7]. Roots are the frontline organs making contact with salt in the soil. They must adapt to the soil salinity to maintain plant growth and uptake of nutrients and water. Salt stress significantly reduces root mass and modifies the distribution of root system architecture components, differentially affecting the growth rate of the primary root and lateral roots and inhibiting lateral root formation. Aboveground tissues are also limited by salt stress, but the molecular mechanisms behind this are less well studied than those for roots. Plants have evolved a series of mechanisms to mitigate the effects of stressful conditions on growth. Therefore, understanding plant tolerance mechanisms is essential for effectively utilizing saline–alkali land and improving crop yields.

High levels of salinity induce ionic stress, osmotic stress, and oxidative stress. Plants employ various physiological and biochemical responses to help maintain ionic homeostasis, proper osmotic potential, and reactive oxygen species (ROS) homeostasis [8].

Great effort has been devoted to understanding the process of salt stress sensing, yielding substantial breakthroughs; however, knowledge of this process remains limited, and it is unclear whether there is a specific Na^+^ receptor. Ionic homeostasis is disrupted under salt stress. Excluding excess Na^+^ is a fundamental approach underpinning plant tolerance of salt stress and has been the focus of considerable research. The SALT OVERLY SENSITIVE (SOS) pathway plays a central role in excluding excess Na^+^ from plant cells. Identification of regulators of the SOS pathway has confirmed the central position of this signaling pathway in the salt-tolerance mechanism. A high level of potassium absorption and a high K^+^/Na^+^ ratio in plant cells are also important for ionic homeostasis [9,10,11,12]. Several K^+^ transporters, such as Arabidopsis K^+^ TRANSPORT 1 (AKT1) and HIGH-AFFINITY K^+^ TRANSPORTER 1 (HKT1), participate in the regulation of the salt stress response. Osmotic homeostasis helps plants maintain proper cellular morphology and ensures the availability of water. High concentrations of soluble salt repress the water potential at the root surface, thereby decreasing water uptake by plants [13] and leading to water deficit and osmotic stress. Ionic and osmotic stresses can also cause secondary stresses in plants, especially the production of toxic ROS, which severely damage cellular structures and reduce the bioactivity of macromolecules [14,15,16]. Various plant hormone signaling pathways respond to salt stress, helping plants to survive under high-salinity conditions by balancing stress tolerance and plant growth. In this review, we briefly summarize how plants respond to salt stress at various levels.

### 1.1. Salt Stress Sensing

Stress sensing is the process by which components (molecules/proteins) known as “sensors” [17] detect changes in the external environment and launch appropriate responses. During saline stress sensing, the calcium signaling response, i.e., the “secondary signaling process” of salt stress, occurs within a timescale of seconds, and excessive Na^+^ starts to be excluded from the root tissue 10 min after exposure to high salinity [18]. These findings suggest that salinity-induced ionic stress is rapidly perceived by plants, initiating the regulation of ionic homeostasis. The detrimental effects of salinity on plant performance often take several hours to manifest after NaCl exposure, suggesting that plants employ different processes for long-term saline-stress sensing.

Sodium ions are initially perceived by sensors localized on the plasma membrane (PM). Saline stress induces ionic stress that results in changes to the calcium status of the cytosol [8]. Thus, the initiation of salt stress signaling transduction is always associated with the regulation of Ca^2+^ channels. Glycosyl inositol phosphorylceramides (GIPCs) are a class of lipids abundant in the PM that collectively function as a monovalent-cation sensor by binding with monocations and regulating ionic-stress-induced Ca^2+^ signaling. Biosynthesis of GIPC is regulated by monocation-induced Ca^2+^ increases 1 (MOCA1), a glucuronosyltransferase involved in GIPC biosynthesis. The *moca1* mutant is sensitive to salt stress and displays a defect in the production of Ca^2+^ spikes induced by excessive Na^+^ [19]. GIPC is thought to be an ionic-specific sensor of salt stress; however, GIPC can also bind K^+^ and Li^+^ [19]. Whether plants have a more specific sodium sensor needs further research.

*AtANNEXIN4* (*AtANN4*) encodes a putative calcium-permeable transporter in *Arabidopsis thaliana* (Arabidopsis) that interacts with SOS3-LIKE CALCIUM-BINDING PROTEIN8 (SCaBP8) and the kinase SOS2 under salt stress to induce a calcium signal at the beginning of salt stress and initiate the SOS pathway, a conserved salt-stress-tolerance signaling pathway [20,21]. SCaBP8 and activated SOS2 form a complex that phosphorylates ANN4 to repress its calcium-permeable transporter activity, thereby creating a specific calcium signal that functions in the long-term salt stress response [20]. This suggests that the long-term salt stress response is different from the short-term stress response at the molecular level.

Increasing evidence indicates that changes in the plant cell wall participate in sensing salt stress [22,23]. Root tissue cells display a radial swelling when plants are treated with high salinity for 6–8 h [24,25], with the integrity of the plant cell wall changing under high-salinity treatment. In Arabidopsis, the PM-localized leucine-rich repeat receptor kinase (LRR-RK) Male Discoverer 1-Interacting Receptor-Like Kinase 2/Leucine-Rich Repeat Kinase Family Protein Induced by Salt Stress (MIK2) [23], as well as FEI1 and FEI2 (two additional leucine-rich repeat receptor kinases), function in perceiving changes in cell wall integrity as well as in the salt stress response [26]. In Arabidopsis, FERONIA (FER), a PM-localized receptor-like kinase, is required for the recovery of root growth when plants are subjected to high salinity. Pectin polysaccharide in the cell wall interacts with the extracellular domain of FER. High salinity weakens the cell wall and disrupts its integrity. FER perceives these changes and regulates calcium channels to mediate calcium signaling, thereby maintaining root cell morphology during growth under salt stress [25]. The content and composition of cell wall lignin are affected when plants are exposed to biotic and abiotic stresses [27]. *SHORT ROOT IN SALT MEDIUM 3* (*RSA3*)*/MURUS3* (*MUR3*)*/KATAMARI1* (*KAM1*) encodes a xyloglucan galactosyltransferase involved in cell wall biosynthesis in Arabidopsis. KAM1 maintains endomembrane organization and prevents damage to plant cells under salt stress by decreasing cellular ROS accumulation and regulating the expression of stress-related genes [22]. These observations indicate that cell wall integrity status and changes in cell wall components under salt stress trigger downstream signaling, stimulating plant responses and initiating salt-tolerance mechanisms to regulate root growth and morphogenesis for adapting to stress conditions. These processes are typically initiated long after the beginning of salt stress, further confirming that the early response of plants to salt stress is different from their response to long-term salt stress and that plants employ different mechanisms for sensing short-term vs. long-term salt stress.

Saline stress induces both ionic stress and osmotic stress, with half of the stress response genes induced by salt stress also being induced by osmotic stress [28]; this suggests that osmotic perception probably contributes to the process of sensing salt stress. Rice (*Oryza sativa*) REDUCED HYPEROSMOLALITY-INDUCED CALCIUM INCREASE 1 (OSCA1) is a channel responsible for increases in internal calcium ion concentration induced by a stimulus in plants and is therefore considered to be the osmotic sensor. The *osca1* mutant shows defective Ca^2+^ signaling under osmotic stress in root cells and guard cells, as well as decreased regulation of transpiration and inhibited root growth under osmotic stress [29,30]. Pei and colleagues recently demonstrated that OSCA1 functions as an osmotic-specific sensor [31]. Calcium signaling in the *osca1* mutant is reduced more greatly under osmotic stress treatment than under NaCl treatment [30]. These findings confirm that salt-induced osmotic sensing differs mechanistically from ionic stress sensing. However, the *osca1.1* mutant is hypersensitive to both sorbitol and NaCl, demonstrating that the osmotic sensor OSCA1.1 indeed contributes to the salt stress response of plants through salinity-induced osmotic perception [32].

### 1.2. Ion Homeostasis Regulation

Maintaining ionic homeostasis in cells is critical for plants in adapting to the presence of excess ions during salt stress. An appropriate ratio of K^+^/Na^+^ is required to maintain low sodium and high potassium levels in the cytoplasm, which prevent cellular damage and nutrient deficiency under salt stress [8,33]. Ionic homeostasis is related to ion transport. Sodium ions are transported into plant cells in a non-selective and non-active manner. No specific transporters responsible for Na^+^ influx have been identified yet in plant cells; rather, the uptake of Na^+^ into the cytoplasm is considered to occur passively. Excessive Na^+^ in the extracellular space induces the generation of an electrochemical potential across the PM, which promotes the movement of Na^+^ across the PM into the cytoplasm. Membrane potential differences (PDs) exhibit characteristic noise under salinity treatment. Upon salt stress, depolarization of membrane PDs to an excitation threshold sets off successive action potentials, leading to further losses of K^+^ and Cl^−^ [34].

Sodium ions are probably transported into the cytoplasm via the K^+^ transport systems. K^+^ UPTAKE 1 (KUP1) is a dual-affinity K^+^ transporter responsible for K^+^ uptake in Arabidopsis [9]. Na^+^ competes with K^+^ for uptake by KUP1, suggesting that the influx of Na^+^ and K^+^ might occur via similar mechanisms. The low-affinity K^+^ transporters AKT1 and HKT1 both mediate the movement of Na^+^ across the PM [33,35,36]. Non-selective cation channels (NSCCs), voltage-independent cation channels (VICs), and non-selective outward-rectifying conductance (NORC) are also involved in the influx of Na^+^ and K^+^ into plant cells [35,37].

Unlike Na^+^ uptake, Na^+^ efflux is an active process. A PM-localized ATPase activated through Na^+^ was identified in the unicellular chrysophyte *Heterosigma akashiwo* [38,39]. PM H^+^-ATPases power Na^+^ exclusion in land plants [40,41]. In Arabidopsis, Na^+^/H^+^ EXCHANGER1 (NHX1) is a tonoplast-membrane-localized Na^+^/H^+^ antiporter responsible for transporting sodium ions from the cytosol into the vacuole [42]. HKT1s are a class of Na^+^ and K^+^ transporters with a stronger affinity for Na^+^ [43]. The xylem-parenchyma-localized transporter AtHKT1;1 is responsible for unloading Na^+^ from xylem vessels to repress the transport of excess Na^+^ from root to shoot tissues [44,45,46].

**Figure 1:**
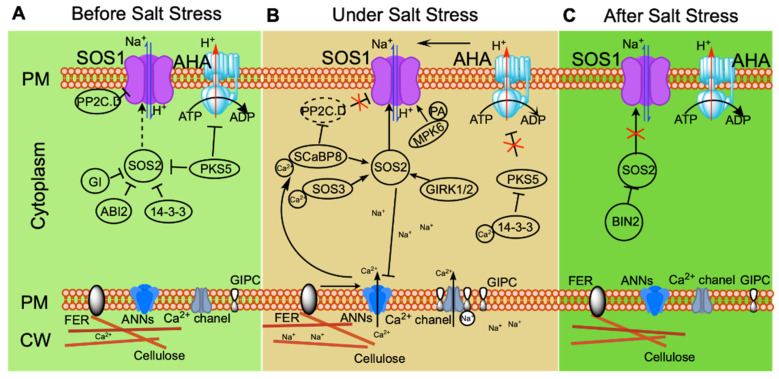
Ionic-stress signaling pathways that maintain ionic homeostasis and thereby help plants to adapt to salt stress. Under non-stress conditions (before salt stress (**A**)), plasma membrane (PM) H^+^-ATPase activity is repressed by PKS5; SOS2 kinase activity is repressed by PKS5, 14-3-3, ABI2, and GI; and SOS1 activity is inhibited by clade D PP2C (PP2C.D). Under salt stress (**B**), GIPC binds Na^+^, inducing an increase in calcium signaling. FER perceives changes in the cell wall under long-term salt stress and mediates calcium signaling. The calcium receptors SOS3 and SCaBP8 bind Ca^2+^, interacting with and activating SOS2, which then phosphorylates SOS1 to activate its Na^+^/H^+^ antiporter activity. Salt stress induces PA accumulation, which promotes the kinase activity of MPK6. MPK6 then phosphorylates SOS1 to enhance the activity of SOS1. At the same time, SCaBP8 inhibits PP2C.D to relieve the inhibition of SOS1 by PP2C.D. ANN modulation of calcium signaling under salt stress positively regulates SCaBP8-activated SOS2; under long-term salt stress, SOS2 phosphorylates ANN4 and represses its Ca^2+^ channel activity, creating a specific calcium signal for long-term stress. After salt stress (**C**), BIN2 phosphorylates SOS2 and inhibits its kinase activity, helping plants to recover from stress.

Na^+^ exclusion from cells is the main approach used by plants to resist salt stress. The Ca^2+^-dependent Na^+^ efflux pathway SOS was identified using genetic screens of Arabidopsis *sos* mutants subjected to salt stress and is the most powerful Na^+^ efflux pathway in plants (Figure 1). The SOS pathway consists of SOS3, SCaBP8, SOS2, and SOS1. SOS3 and SCaBP8 are helix E–loop–helix F (EF-hand) Ca^2+^-binding proteins responsible for sensing and decoding calcium signals in the cytosol stimulated by excessive salinity [21,47,48,49]. SOS3 functions primarily in roots, whereas SCaBP8 functions primarily in shoots [21,49]. Both proteins interact with SOS2, the most critical serine/threonine protein kinase in the SOS pathway, activating its kinase activity. Further, SOS2 is recruited to the PM by SOS3 and SCaBP8 for phosphorylation of the Na^+^/H^+^ antiporter SOS1 [21,50,51,52,53]. SOS1 is the most important determinant of Na^+^ transport out of the cytoplasm to the apoplast, which is promoted by a PM H^+^-ATPase-generated proton gradient [52,54]. The C-terminus of SOS1 is an intracellular domain containing an autoinhibitory domain, while the N-terminus is a transmembrane domain. The transport activity of SOS1 is autoinhibited by intermolecular interaction between these two domains [55]. SOS2 phosphorylates the 1136th and 1138th serines in the C-terminal autoinhibitory domain of SOS1, which relieves the autoinhibition of SOS1, resulting in SOS1 activation [56,57].

Regulation of the SOS pathway has been the subject of extensive research, showing among other things that SOS2 plays an irreplaceable role in the pathway. In Arabidopsis, SOS2 kinase is induced specifically by sodium, but not by potassium or mannitol [51]. Structural analysis suggests that the N-terminus of SOS2 is a kinase domain, while its C-terminus is a regulatory domain that interacts with the kinase domain to cause self-inhibition. A 21-amino-acid sequence in the regulatory domain of SOS2, named the FISL motif (owing to the presence of the conserved residues A, F, I, S, L), is responsible for the binding of SOS2 to SOS3. The kinase activity of SOS2 requires coordinated release of the self-inhibitory FISL motif and the activation loop [58,59]. Under normal conditions, SOS2 kinase activity is inhibited through phosphorylation by PROTEIN KINASE SOS2-LIKE 5 (PKS5). 14-3-3 proteins repress SOS2 activity, and this repression is enhanced by the phosphorylation of SOS2 by PKS5 [60,61]. GIGANTEA (GI) also functions as a repressor of SOS2 kinase activity through interaction with SOS2. When plants experience saline stress, inhibition of SOS2 activity is released by degradation of GI and 14-3-3 through a 26S proteasome pathway [61,62]. Ca^2+^ signaling is stimulated under salt stress. This is perceived by 14-3-3 proteins that interact with PKS5 and suppress its kinase activity, further relieving the repression of SOS2 kinase activity by 14-3-3 and PKS5 and initiating Na^+^ efflux in the SOS pathway [60,61]. When salt stress is removed, SOS2 activity is inhibited through phosphorylation by BRASSINOSTEROID-INSENSITIVE 2 (BIN2), a protein kinase functioning as a negative regulator in the brassinosteroid signaling pathway; plants attenuate stress responses and focus on growth [63]. The protein phosphatase ABSCISIC ACID-INSENSITIVE 2 (ABI2) also inhibits SOS2 activity through an unknown molecular mechanism; *abi2-1*, a dominant-negative mutant of *ABI2*, is insensitive to NaCl compared with the wild type [64]. GEMINIVIRUS REP INTERACTION KINASE1 (GRIK1) and GRIK2 function as positive regulators of SOS2 kinase activity by phosphorylating the activation loop of SOS2 to activate its kinase activity [65]. These regulations of SOS2 illustrate the important role of SOS2 at the center of the SOS pathway.

In addition, SOS2 also functions in other pathways. However, the mechanism regulating SOS2 kinase activity and adjusting it between salt stress and non-salt-stress conditions is not clearly understood. Salt stress induces ROS stress [1], which in turn modulates the transcription level of SOS1 [66], indicating that the SOS pathway is modulated by ROS signals [1,67]. SOS2 interacts with CATALASE 2 (CAT2) and CAT3, two crucial regulators of H_2_O_2_ signaling, suggesting that SOS2 serves as a node connecting H_2_O_2_ signaling and salt stress responses [68]. Ethylene-insensitive 3 (EIN3) is phosphorylated by SOS2, and ethylene-related gene expression is thereby enhanced; therefore, salt stress and ethylene signaling are linked by SOS2 [69]. In summary, SOS2 functions as a connector linking the SOS pathway with other signaling pathways.

Various regulators of SOS1 have also been identified in recent years. Salt stress increases phosphatidic acid (PA) accumulation in the PM. PA binds to MITOGEN-ACTIVATED PROTEIN KINASE 6 (MPK6) and activates its kinase activity; the C-terminus of SOS1 is phosphorylated by PA-activated MPK6, in turn activating the Na^+^/H^+^ antiporter activity of SOS1 [70]. Two Clade D protein phosphatases 2C, D6 and D7 (PP2C.D6 D7), interact with SOS1 to repress its activity in a process dependent on their phosphatase activity under non-stress conditions. SCaBP8 perceives salinity-induced calcium signaling and inhibits the phosphatase activity of PP2C.D6 and D7 under salt stress, simultaneously regulating the subcellular localization of PP2C.D6, which mediates PP2C.D6 moving to the cytoplasm from the plasma membrane in turn to release SOS1 activity [71]. Identification of more and more regulators controlling the activity of the SOS pathway at different stages of stress confirms that the SOS pathway plays a central role in the regulating of ionic homeostasis (Figure 1).

In conclusion, in the absence of salt stress, the SOS pathway is maintained in a low-activity state. Maintenance of this state depends on the repression of SOS2 kinase activity by PKS5, 14-3-3, GI, and ABI2; inhibition of SOS1 by PP2C clade D; and suppression of PM H^+^-ATPase by PKS5 (Figure 1A). When plants experience salt stress, GIPC binds to Na^+^, increasing calcium signaling. Calcium receptors SOS3 and SCaBP8 bind the intracellular Ca^2+^ and interact with and activate SOS2, which phosphorylates SOS1 to activate its Na^+^/H^+^ antiporter activity. MPK6 is activated by PA induced in response to salt stress, further phosphorylating SOS1 and promoting its activity. Simultaneously, SCaBP8 relieves the inhibition of SOS1 by repressing the protein phosphatase activity of PP2C.D. Inhibition of H^+^-ATPase (AHA) by PKS5 is relieved by 14-3-3, creating a proton gradient across the PM that drives Na^+^/H^+^ antiporter SOS1 activity. FER perceives changes in the cell wall under salt stress and mediates calcium signaling for long-term stress. ANNEXINs (ANNs) modulate calcium signaling under salt stress, promoting activation of SOS2 activity by SCaBP8; SOS2 phosphorylates ANN4 and represses its Ca^2+^ channel activity, creating a specific calcium signaling cascade for long-term salt stress (Figure 1B). After salt stress is relieved, BIN2 phosphorylates SOS2 to inhibit its kinase activity, helping plants to recover from stress (Figure 1C). The capacity of plants to exclude Na^+^ needs to be appropriately regulated at different stages of stress for the maintenance of ionic homeostasis.

The SOS pathway is involved in potassium uptake and is crucial for regulating Na^+^/K^+^ homeostasis in plants [49]. AKT1 is an important potassium channel contributing to K^+^ influx transport. Salt stress decreases the transport activity of AKT1 [72]. SCaBP8 interacts with the C-terminus of AKT1 to inhibit its K^+^ transport activity [73]. The Arabidopsis *sos1* mutant shows significantly reduced transcript levels of *AtAKT1, AtHKT1;1*, and STELAR K^+^ OUTWARD RECTIFIER (*AtSKOR*; encoding the single outward Shaker K^+^ channel) in root tissues compared with the wild type, as well as elevated accumulation of Na^+^ in root cells and decreased K^+^ uptake and transport, ultimately leading to suppression of plant growth [74]. Salinity stress also influences K^+^ levels by regulating another potassium transporter, TONOPLAST-LOCALIZED K^+^ CHANNEL1 (TPK1) [75]. CALCIUM-DEPENDENT PROTEIN KINASE (CDPK) phosphorylates TPK1 and activates K^+^ influx under salt stress [76]. Nonetheless, how plants actively regulate potassium uptake, including whether SOS signaling pathway elements directly regulate potassium uptake under salt stress, remains unclear.

### 1.3. Osmotic Homeostasis

Osmotic stress induced by salinity leads to various transient biophysical changes, such as shrinkage of the PM, decreases in turgor pressure in the cell, and physical changes to the cell wall [77]. Many proteins play essential roles in regulating salt-induced osmotic stress responses [8,78]. The osmotic-stress-stimulated Ca^2+^ channel OSCA1 is considered to be a hyperosmotic stress sensor [29,30]; however, the *osca1* mutant shows no significant phenotypic differences from the wild type under salt stress. Therefore, whether OSCA1 is involved in salt-induced osmotic stress responses needs to be further researched.

The plastidial EXCHANGE ANTIPORTER 1 (KEA1), KEA2, and KEA3 regulate Ca^2+^ signaling induced by rapid hyperosmotic stress, and *kea* mutants are defective in Ca^2+^ signaling [79]. Therefore, KEA1/2/3 are thought to function as sensors of osmotic stress that regulate increases in Ca^2+^ levels under stress conditions. MscS-LIKE8 (MSL8), a PM-localized mechanosensitive (stretch-activated) ion channel in Arabidopsis pollen, is a sensor of hyperosmotic-stress-induced membrane tension. MSL8 promotes pollen survival under the hyperosmotic shock of rehydration [80]. The Ca^2+^-responsive phospholipid-binding copine protein BONZAI1 (BON1) plays a critical role in osmotic stress regulation by positively regulating Ca^2+^ signaling. Defects in BON1 disrupt Ca^2+^ signaling in the cytosol in response to osmotic stress [81]. Whether these osmotic sensors participate in salt-stress-induced osmotic stress sensing and regulation requires further investigation.

MAPK cascades are involved in signal transduction in response to both salt and osmotic stress. Under salt stress, PA-activated MPK6 phosphorylates SOS1, improving its Na^+^/H^+^ antiporter activity in Arabidopsis [69]. PA-mediated activation of MAPK signaling cascades under hyperosmotic stress caused by high salt and desiccation has also been observed in *Asterochloris erici* [82]. The MKK4-MPK3 and MKKK20-MPK6 pathways participate in the osmotic stress responses. *mkk4* and *mkkk20* mutants exhibit increased sensitivity to salt stress and increased water loss under dehydration conditions compared with the wild type [83,84]. Under osmotic stress treatments, mRNA levels of *MAPKKK, MAPKK*, and *MAPK* genes are upregulated, increasing the biosynthesis and accumulation of osmolytes [85,86].

Water uptake capacity is destroyed by salt stress, leading to dehydration and changes in cell turgor and thus to osmotic stress. Under high-salinity conditions, endogenous abscisic acid (ABA) levels increase to mediate stomatal closure and further regulate osmotic homeostasis [87]. ABA functions as a key link between salt stress and osmotic stress responses. SUCROSE NON-FERMENTING1-RELATED PROTEIN KINASES (SnRKs) are the central components regulating the ABA signaling pathway, and almost all SnRK2s are activated by osmotic stress. ABA binds to its receptors and participates in relieving the inhibition of SnRK2.2/3/6 kinase activity by clade A PP2Cs, initiating an ABA-responsive element (ABRE)-binding protein/ABRE-binding factor (AREB/ABF) signaling pathway to regulate osmotic stress and drought stress tolerance [8,79,88]. The SnRK2-AREB/ABF regulatory network modulates starch degradation in leaves through β-AMYLASE1 (BAM1)/α-AMYLASE3 (AMY3), which is also crucial for osmotic stress tolerance in plants [89]. SnRKs function in osmotic stress regulation in an ABA-independent manner. VARICOSE (VCS) is an mRNA decapping activator that belongs to the osmotic-stress-activated subclass I SnRK2s, which are involved in the regulation of mRNA populations under osmotic stress [88].

ABA also participates in the salt stress response through other regulators. FER functions in response to cell wall defects induced by long-term salt stress. ABI2, an important protein phosphatase that functions in ABA signaling, dephosphorylates FER, which senses defects in the cell wall. ABA regulates the kinase activity of FER via ABI2 [90], and, at high levels, helps prevent decreases in photosynthesis resulting from salt stress [91]. In all studies demonstrating crosstalk between the salt stress response and ABA signaling, high salinity increased endogenous ABA levels. Whether a direct interaction or a regulatory relationship exists between the central components of the salt stress and ABA signaling pathways remains unclear.

Common osmotic response pathways (both long-term and short-term) result in the biosynthesis and accumulation of compatible osmolytes, which can correct the cellular osmotic potential in cells and stabilize proteins and cellular structures and morphology. This is an adaptive strategy that is not specifically induced by salt stress. Compatible osmolytes prevent water loss to resist short-term osmotic stress and increase cellular turgor and cellular expansion to cope with long-term osmotic stress [1,92,93,94,95,96]. Many compatible osmolytes biosynthesized under salt stress also accumulate under other stresses, such as drought and cold stresses, and their biosynthesis is partly species- and tissue-specific [1,97,98,99,100].

Salt stress induces the accumulation of several types of osmolytes, including charged metabolites, polyols, sugars, and complex sugars. Charged metabolites include β-alanine betaine, choline-O-sulfate, glycine betaine, hydroxyproline, dimethyl sulfonium propionate (DMSP), proline, and putrescine [101,102,103,104]. Polyols include glucosylglycerol, glycerol, myoinositol, ononitol, pinitol, mannitol, and sorbitol [105,106,107,108,109,110,111]. Sugars include fructose and sucrose as well as the complex sugars trehalose, fructans, and raffinose. Ions such as K^+^ also function in osmotic regulation [111,112,113,114,115,116,117,118]. These osmolytes also act as signals of ABA accumulation under salt stress, regulating target gene expression [119].

### 1.4. Regulation of Oxidative Stress Responses

**Figure 2:**
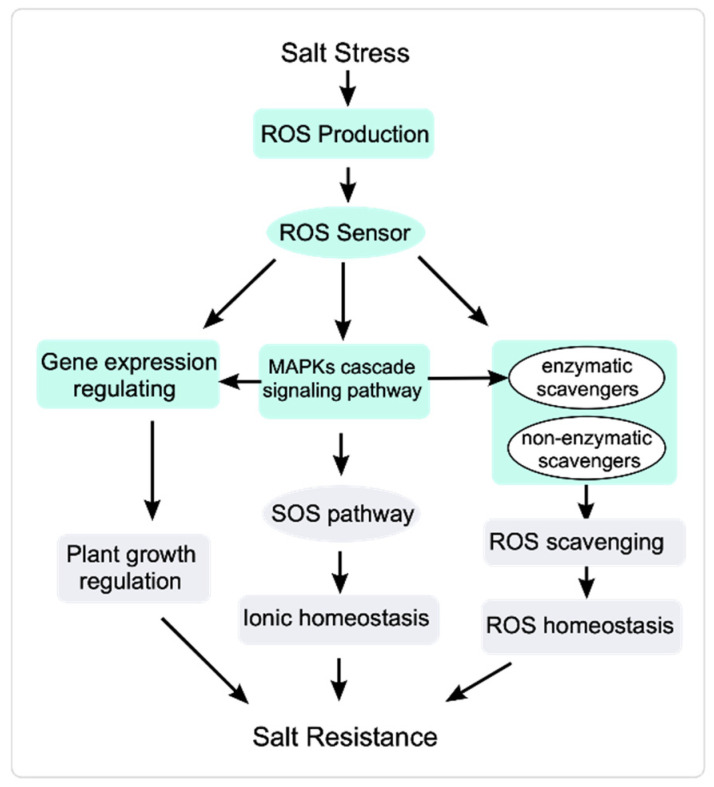
ROS signal transduction response to salt stress. Salt stress induces a rapid increase in ROS accumulation. Sensors perceive the elevated ROS and transduce the ROS signal to stimulate plant responses. MAPK signaling cascades receive ROS signals and regulate the activity of the SOS pathway and ROS scavengers to modulate ionic homeostasis and ROS homeostasis, respectively. MAPKs also regulate gene expression to modulate plant growth under salt stress.

Oxidative stress is a secondary stress stimulated by salt stress in plants, with salt stress rapidly inducing the accumulation of toxic ROS and oxidative damage [8]. Although high concentrations of ROS are damaging to plants, ROS function as signaling molecules at low concentrations [1,120]. Plant cells perceive high ROS levels and rapidly initiate their elimination by scavenging ROS and inducing adaptive responses [11,121,122] (Figure 2). In plant cells, ROS comprise free radicals and non-radicals. The free radicals include superoxide radical (O_2_^•−^), hydroxyl radical (OH^•^), alkoxyl radical (RO^•^), and peroxyl radical (ROO^•^); non-radical ROS are singlet oxygen (^1^O_2_), hydrogen peroxide (H_2_O_2_), hypochlorous acid (HOCl), and excited carbonyl (RO*). The predominant ROS in plant cells are ^1^O_2_, H_2_O_2_, O_2_^•−^, and ^•^OH [123,124].

MAPKs are involved in regulating ROS homeostasis [125]. The receptor-like kinase SALT INTOLERANCE 1 (SIT1) activates MAPK3 and MAPK6 and regulates the homeostasis of ROS and ethylene to inhibit plant growth and even promote death during the salt stress response [126]. Salt stress increases *ZmMPK5/17* levels and regulates oxidative homeostasis to help maize (*Zea mays*) plants cope with salt stress [127,128]. In Arabidopsis, MPK3/6 positively regulate salt stress responses by phosphorylating HEAT SHOCK FACTOR A4A (HSFA4A) and modulating ROS homeostasis [129]. ROS scavenging is important for regulating ROS homeostasis. MAPKs also activate ROS scavengers and regulate the expression of ROS-responsive genes to regulate oxidative homeostasis [130]. Transcriptome analysis of *mekk1*, *mkk1/2*, and *mpk4* mutants shows that the MEKK1-MKK1 and MKK2-MPK4 regulatory pathways modulate the activity of ROS scavenging enzymes to help maintain ROS homeostasis [131] (Figure 2).

**Figure 3:**
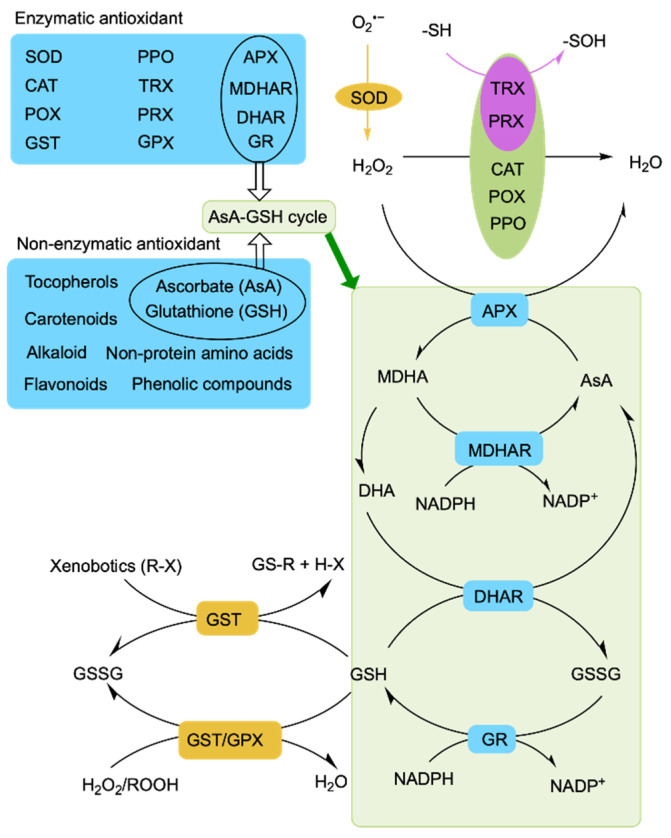
Outline of antioxidant defense mechanisms in plants. SOD, superoxide dismutase; CAT, catalase; POX, peroxidase; AsA, ascorbate; DHA, dehydroascorbate; GSSG, oxidized glutathione; GSH, reduced glutathione; APX, ascorbate peroxidase; MDHA, monodehydroascorbate; MDHAR, monodehydroascorbate reductase; DHAR, dehydroascorbate reductase; GR, glutathione reductase; GST, glutathione S-transferase; GPX, glutathione peroxidase; PPO, polyphenol oxidase; PRX, peroxiredoxin; TRX, thioredoxin; NADPH, nicotinamide adenine dinucleotide phosphate; O, oxygen; H_2_O_2_, hydrogen peroxide; O2^•−^, superoxide radical; R, aliphatic, aromatic, or heterocyclic group; X, sulfate, nitrite, or halide group; ROOH, hydroperoxides; -SH, thiolate; -SOH, sulfenic acid.

ROS levels must be strictly regulated to avoid their destructive effects on plant cells, so antioxidants are employed to modulate ROS metabolism [1]. Plant cells initiate rapid regulatory mechanisms to scavenge ROS when high ROS levels are sensed under environmental stress [11]. Many enzymatic and non-enzymatic antioxidant scavengers help to prevent damage induced by ROS in plants under salt stress [132,133,134]. Enzymatic ROS scavengers include ascorbate peroxidase (APX), catalase (CAT), polyphenol oxidase (PPO), dehydroascorbate reductase (DHAR), peroxidase (POX), glutathione peroxidase (GPX), peroxiredoxins (PRXs), thioredoxin (TRX), glutathione peroxidase (GR), glutathione S-transferase (GST), monodehydroascorbate reductase (MDHAR), and superoxide dismutase (SOD) [132,135,136,137,138,139,140]. Ascorbate (AsA), glutathione (GSH), alkaloids, carotenoids, flavonoids, phenolic compounds, non-protein amino acids, and tocopherol function as non-enzymatic scavengers [141,142,143,144,145,146,147] (Figure 3).

Among antioxidant enzymes, SOD plays an important role as the first line of defense and converts O_2_^•−^ into H_2_O_2_. The H_2_O_2_ is further converted into H_2_O and O_2_ by the H_2_O_2_ enzymes CAT, APX, and GPX [148] (Figure 3). CAT is an important enzyme that rapidly decomposes H_2_O_2_ into H_2_O and O_2_ without any reducing equivalent [149]. *NCA1* (*NO CATALASE ACTIVITY1*) encodes a protein that can interact with CAT2 and enhance its catalase activity, thereby promoting CAT2 regulation of ROS homeostasis through scavenging cellular hydrogen peroxide under abiotic stress [132]. The *nca1-3* mutant is hypersensitive to various abiotic stresses including saline stress. Heat shock protein (Hsp) 17.6CII, a chaperone protein, also activates CAT2 together with NCA1 to increase the abiotic stress tolerance of Arabidopsis [134]. In rice, the receptor-like kinase Salt Tolerance Receptor-like cytoplasmic Kinase 1 (STRK1) phosphorylates the CAT CatC to increase salt tolerance [148]. The AsA-GSH cycle comprises ASA, GSH, and four antioxidant enzymes (APX, DHAR, GR, and MDHAR), playing a significant role in the regulation of ROS homeostasis through detoxifying H_2_O_2_ [150]. Alkaloids can inhibit the oxidation induced by H_2_O_2_ and scavenge free radicals [151]. Tocopherol mainly scavenges ^1^O_2_ and ^•^OH to maintain photosynthesis and protect chloroplasts [152]. Carotenoids, flavonoids, and phenolic acids regulate ROS homeostasis by scavenging free radicals [153,154,155,156]. All of this evidence suggests that plants actively engage in regulatory mechanisms to cope with salt-stress-induced oxidative stress and transduce stress signals by controlling ROS homeostasis.

### 1.5. Phytohormonal Responses to Salt Stress

Besides transporting excess Na^+^ out of cells, plants must also adjust their growth to adapt to salt stress or any other adverse environmental conditions. These growth adjustments require regulation by various plant hormones. In addition, salt stress elicits the responses of several plant hormones directly.

Auxin plays crucial roles in several aspects of plant development, such as shoot and root architecture, maintenance of meristem activity, and leaf morphogenesis. Many studies have demonstrated that auxin levels decrease under salt stress [157,158,159,160], but the processes regulating auxin biosynthesis under saline stress appear to be complex. Endogenous auxin biosynthesis is regulated by the YUCCA family of flavin monooxygenases (YUC). Expression of *CsYUC10a* and *CsYUC11* decreases in cucumber (*Cucumis sativus*) under salt stress. However, the expression of *YUC6* in poplar and potato and *CsYUC11* in Arabidopsis enhances tolerance of salt stress [160,161,162], pointing to a complicated regulation process for auxin biosynthesis under saline stress. *TRANSPORT INHIBITOR RESPONSE 1* (*TIR1*), encoding an auxin receptor, and *AUXIN SIGNALLING F-BOX* (*AFB*) are also downregulated under salt stress [163], leading to changes in the expression patterns of the auxin transporter genes *AUXIN-RESISTANT1* (*AUX1*), *PIN-FORMED1* (*PIN1*), and *PIN2* [164]. These results illustrate how plants adjust their auxin response and auxin transport to adapt to saline stress, possibly as a means of balancing plant growth and stress tolerance.

Brassinosteroids (BRs) are plant hormones that promote plant growth. The receptor BRASSINOSTEROID INSENSITIVE 1 (BRI1) recognizes the presence of BR through a series of phosphorylation cascade reactions among BR-related kinases. The key transcription factors BRASSINAZOLE RESISTANT 1 (BZR1) and BRI1-EMSSUPPRESSOR 1 (BES1) in the BR signaling pathway are then released to initiate expression of BR-responsive genes [165]. Increasing evidence indicates that BRs are involved in salt stress tolerance: The salinity tolerance of plants is enhanced by the exogenous application of BRs [166,167], and mutants of BR-biosynthesis-related genes and positive regulators of BR signaling are more sensitive to salt stress than wild-type plants [168,169,170,171]. These findings suggest that BRs positively regulate plant tolerance to salt stress. BIN2, a negative regulator of BR signaling, directly interacts with and phosphorylates SOS2, inhibiting its kinase activity and thereby downregulating the SOS pathway during the recovery stage after salt stress [63]. This suggests that BR signaling undergoes direct crosstalk with the SOS pathway via their central elements to modulate responses to different stages of salt stress.

Ethylene levels rise under salt stress, with salt stress upregulating the *1-Aminocyclopropane-1-carboxylic acid* (*ACC*) *synthase* (*ACS*) ethylene biosynthesis genes to different degrees. Treatment with the ethylene precursor ACC enhances salt tolerance in plants [163,172,173]. Ethylene signaling is also involved in the salt stress response. Most mutants of positive regulators of ethylene signaling, such as *etr2* (*ethylene response 2*)*, ein2* (*ethylene insensitive 2*)*,* and *ein3/eil1* (*ein3-like 1*), display increased sensitivity to salt stress, whereas *ctr1* (*constitutive triple response 1*), a mutant of a negative regulator of ethylene signaling, is insensitive to salt stress [171]. These findings demonstrate that both ethylene and the ethylene signaling pathway promote salt tolerance in plants.

Salicylic acid (SA) at appropriate levels also promotes salt tolerance in plants. Oxidative toxicity and osmotic stress are both alleviated by the exogenous application of SA, but this positive effect requires a specific dosage range. Seed germination under salt stress is enhanced by the application of less than 50 μM SA and repressed by the application of more than 100 μM SA [174,175].

Gibberellin (GA) also functions as a growth-promoting hormone in plants. However, plants appear to actively downregulate GA signaling and retard their growth to aid survival under salt stress. Salt stress decreases the levels of DOMINANT SUPPRESSOR OF KAR2 (OsDSK2a), a positive regulator of GA signaling, which reduces active GA levels and slows plant growth [176]. The overexpression of genes encoding negative regulators of GA accumulation, such as *CYP71D8* (*CYP, Cytochrome P450*) in rice and *PtCYP714A3* in poplar *(Populus trichocarpa)*, promotes salt tolerance by inhibiting plant growth [177,178]. This implies that GA plays a crucial role in defending plants from salt stress by modulating the balance between growth and stress tolerance.

In summary, studies into the roles of plant hormones in plant responses to salt stress have revealed that balancing plant growth and stress tolerance is crucial for plant survival under salt stress. Cooperative regulation by different plant hormones is essential for maintaining this balance.

### 1.6. Photosynthesis under Salt Stress

Salinity stress destroys the photopigment system and decreases chlorophyll production, leading to severe inhibition of photosynthesis [179]. Both nitrogen and magnesium ions (Mg^2+^) are essential for chlorophyll biosynthesis [180], and salt stress seriously reduces their uptake [181,182], inhibiting chlorophyll biosynthesis. A decrease in stomatal number and an increase in closed stomata under high salt concentrations leads to decreases in CO_2_ absorption, further inhibiting photosynthesis [183,184]. Stomatal density in the adaxial part of phyllodes is significantly lower in *Acacia auriculiformis* under seawater-induced salinity stress compared with that in salt-free control plants [183]. Moreover, salt stress significantly inhibits photosystem II (PSII), which is crucial for light energy conversion and photosynthetic efficiency [185].

The ultrastructure of chloroplasts is affected by salt stress. Salinity stress causes swollen chloroplasts in rice mesophyll cells [186]. In wheat (*Triticum aestivum*), the granum thylakoids of chloroplasts are loosely arranged with a slim spindle shape under 200 mM NaCl compared with non-stress conditions [187]. Salinity stress also results in the swelling of thylakoids and the accumulation of starch granules in *Thellungiella salsuginea*, which helps maintain osmotic equilibrium [188]. Increasing chloroplast number helps halophytes to overcome stomatal limitations induced by salinity stress [189].

Photosynthetic pigments harvest light energy for conversion to chemical energy in the photosystem. Salt stress damages photosynthetic pigments. For example, contents of chlorophyll a, chlorophyll b, and total chlorophyll are reduced in *Acacia auriculiformis* plants under seawater treatment [190]. In *Nicotiana benthamiana*, intermediates of chlorophyll biosynthesis such as Mg-photoporphyrin-IX, protoporphyrin-IX, and protochlorophyllide show reduced abundance under salinity stress [191].

Parameters determining photosynthetic efficiency, including the quantum yield of PSII, activity of PSII, maximum quantum efficiency of PSII photochemistry (*F*_v_/*F*_m_), electron transport (QY), photochemical quenching (qp), non-photochemical quenching (NPQ), and linear electron transport rate (ETR), are significantly altered under salt stress [192]. Many key elements of photosynthesis are inhibited by salinity stress. Salt affects the amount of PSI proteins, with a significant decrease in the amount of PsaA protein observed under NaCl treatment and decreases in the amounts of PsaF and PsaK seen when PEG is applied in combination with salt [193]. Salt stress reduces the activity of ribose 5-phosphate by 55% [194], whereas severe salt stress decreases the regeneration of ribulose 1,5-bisphosphate and carboxylase/oxygenase (RuBisCo), resulting in impaired PSII electron transport [195]. Glyceraldehyde 3-phosphate dehydrogenase phosphoglycerate kinase and phosphofructokinase are also inhibited by salt stress [196].

In conclusion, salt stress leads to lower stomatal conductance and reduced photosynthetic rate, chlorophyll content, and activity of key enzymes, significantly reducing the photosynthetic capacity of plants.

### 1.7. Transcription Factors in Salt Stress Response

Many transcription factors, such as MYB proteins, contribute to salt tolerance. MYB49 positively regulates salt tolerance by upregulating genes encoding peroxidases and late embryogenesis abundant proteins [197]. MYB44 also positively regulates salt tolerance by regulating the expression of *PP2C* genes [198]. RING zinc finger proteins improve salt stress tolerance by regulating ion homeostasis and ROS scavenging. SALT TOLERANCE RING FINGER1 (AtSTRF1), the rice RING-H2-type zinc finger protein OsSIRH2-14, and RING FINGER PROTEIN V6 (OsRFPv6) also positively regulate the salt tolerance of plants [199,200,201].

### 1.8. Implications for Crop Improvement

The mining of important functional genes facilitates molecular breeding, and the development of abiotic stress tolerance, especially saline–alkali-tolerant crop varieties, will allow utilization of the large, and increasing, area of saline–alkali land. Increasing numbers of important regulators of abiotic stress responses have been identified in crops using genome-wide association studies (GWASs) and quantitative trait loci (QTLs). In maize, ZmHAK4 (HIGH AFFINITY K^+^4) confers the capacity for shoot Na^+^ exclusion and salt tolerance [202]; ZmVPP1 (VACUOLAR-TYPE H^+^-PYROPHOSPHATASE) contributes to drought tolerance [203]; and ZmSRO1d (SIMILAR TO RCD-ONEs1d) helps plants to tolerate drought by regulating ROS levels in guard cells to regulate stomatal closure [204]. Alkaline tolerance 1 (AT1) in sorghum can inhibit plant alkali tolerance by negatively regulating the H_2_O_2_ transporter; knockout of *AT1* through gene editing significantly improves the alkali tolerance of crops [205]. These studies suggest the importance of considering oxidative stress regulation in the development of abiotic-stress-tolerant crop cultivars. HKT1 was identified as a major regulator of salt stress tolerance in rice, wheat, and maize through QTL analysis, and marker-assisted breeding of wheat has been developed for increased yield in saline soils [206,207,208,209]. Genes encoding components of the SOS signaling pathway also contribute to phenotypic variation in salt tolerance in maize and tomato [210,211].

At present, the most important breeding methods are direct screening based on phenotypic observation combined with genetic experiments, and molecular marker genotypes [206]. With the rapid development of artificial intelligence (AI), the integration of genomics, phenomics, and environmental factors will play an important role in future breeding [212]. AI can judge data faster and more accurately than humans and precisely predict phenotypes through model training using data collected from multiple sources, including spatiotemporal omics (genomics, phenomics, and enviromics across time and space) [213]. AI technology combined with high-throughput genomics and phenomics has been applied effectively in crop breeding programs [214,215,216]. AI-assisted high-throughput phenotypic systems can effectively and accurately predict phenotypes in maize, wheat, and cassava (*Manihot esculenta* Crantz) [217,218,219]. AI breeding will greatly help scientists select and develop new crop cultivars that are tolerant of stress. However, AI breeding requires the collaboration of plant science, breeding science, bioinformatics, and computer engineering.

## 2. Conclusions and Perspectives

Plant survival under salt stress is a complex process involving crosstalk among several signaling pathways, including osmotic signaling, ROS signaling, and ABA signaling. During the past two decades, numerous studies have focused on the mechanisms regulating ionic homeostasis under salt stress. The SOS pathway has been studied extensively and found to be the most important signaling pathway regulating ionic homeostasis. Ca^2+^ signaling plays an essential role in the transduction of stress signals and serves as a link between different signaling pathways. Phosphorylation is the most important process regulating signaling activity, and kinases are the most important enzymes that perform these regulatory roles. However, many questions remain to be resolved.

First, the mechanism of salt stress sensing requires further study. Plant processes for sensing various ionic stresses appear to differ, prompting the need to identify a specific Na^+^ sensor in plants. Whether more specific sodium receptors exist and whether sodium is perceived extracellularly or intracellularly remain to be explored. Second, much research is needed in order to apply our knowledge of salt-tolerance mechanisms to the development of crops to increase their survival under high salinity. All mutants of SOS genes show a sensitive phenotype in Arabidopsis and other crops [210,211]; however, overexpressing SOS pathway genes only slightly increases plant salt tolerance [220,221]. It is likely that other factors interacting with *SOS* genes during the salt stress response in plants and other salt-tolerance regulators remain to be identified. Finally, for breeding salt-tolerant crop cultivars, multi-omics technologies combined with gene editing will likely serve as efficient tools and lead to wide achievements.

Halophytes are plants that can grow and reproduce under high-salinity conditions (>200 mM NaCl). In addition to sharing salt-tolerance mechanisms with glycophytic plants, halophytes also have evolved specific manners of adapting to high-salinity conditions. For instance, the capacity for osmotic adjustment of halophytes is greater than that of glycophytes [222,223]. Secretion of salt by epidermal bladder cells (EBCs) of halophytes is another specific salt-tolerance mechanism developed by some halophytic species such as *Chenopodium quinoa* [224]. Such specialized salt-tolerance mechanisms will be important for the cultivation of new salt-tolerant crop cultivars.

## Data Availability

Not applicable.
